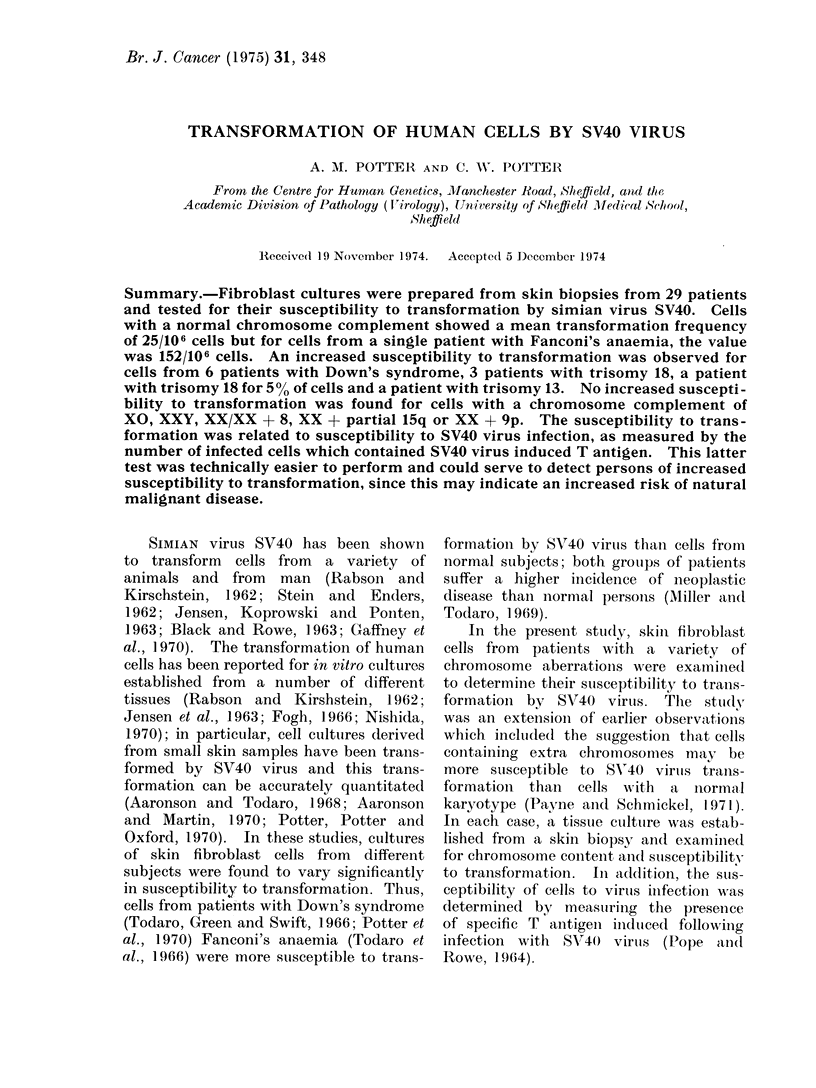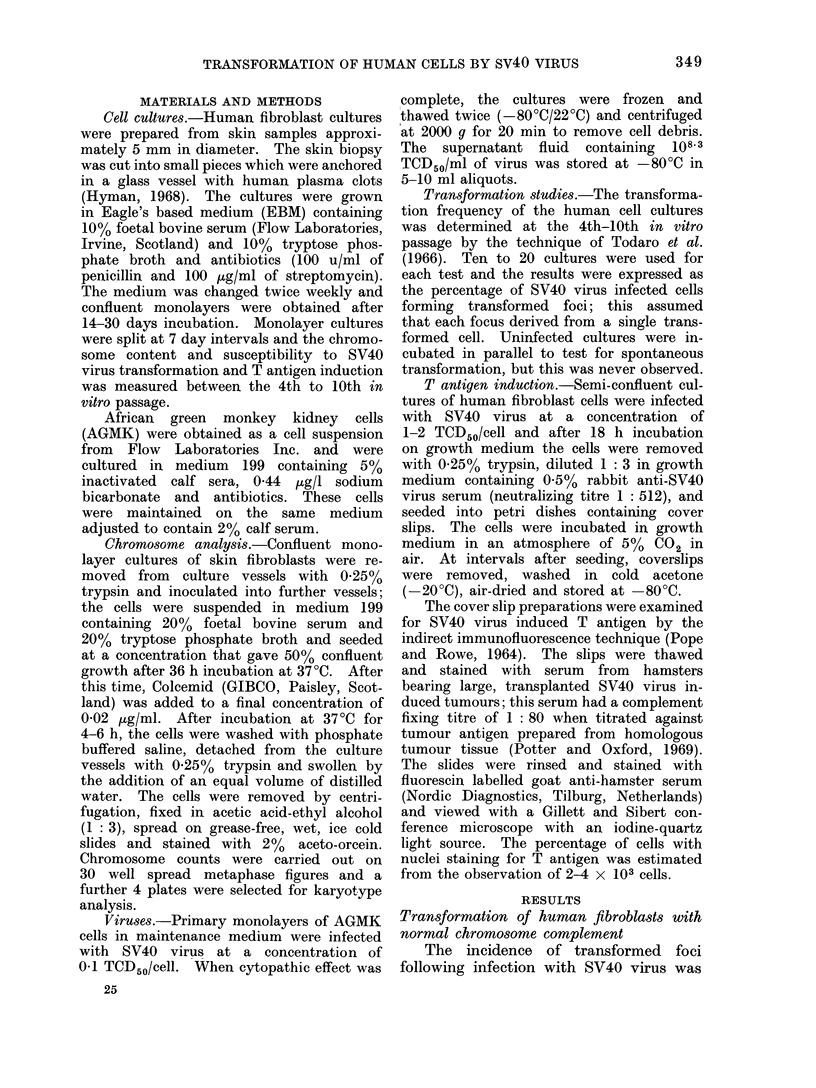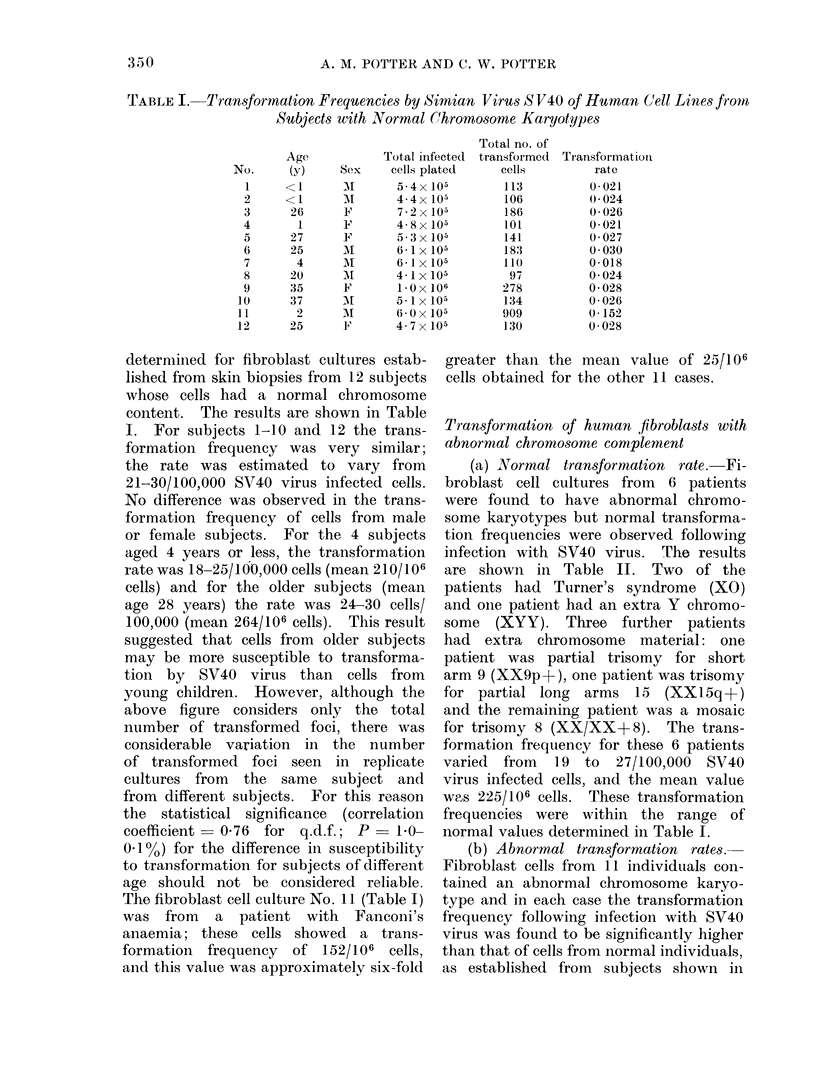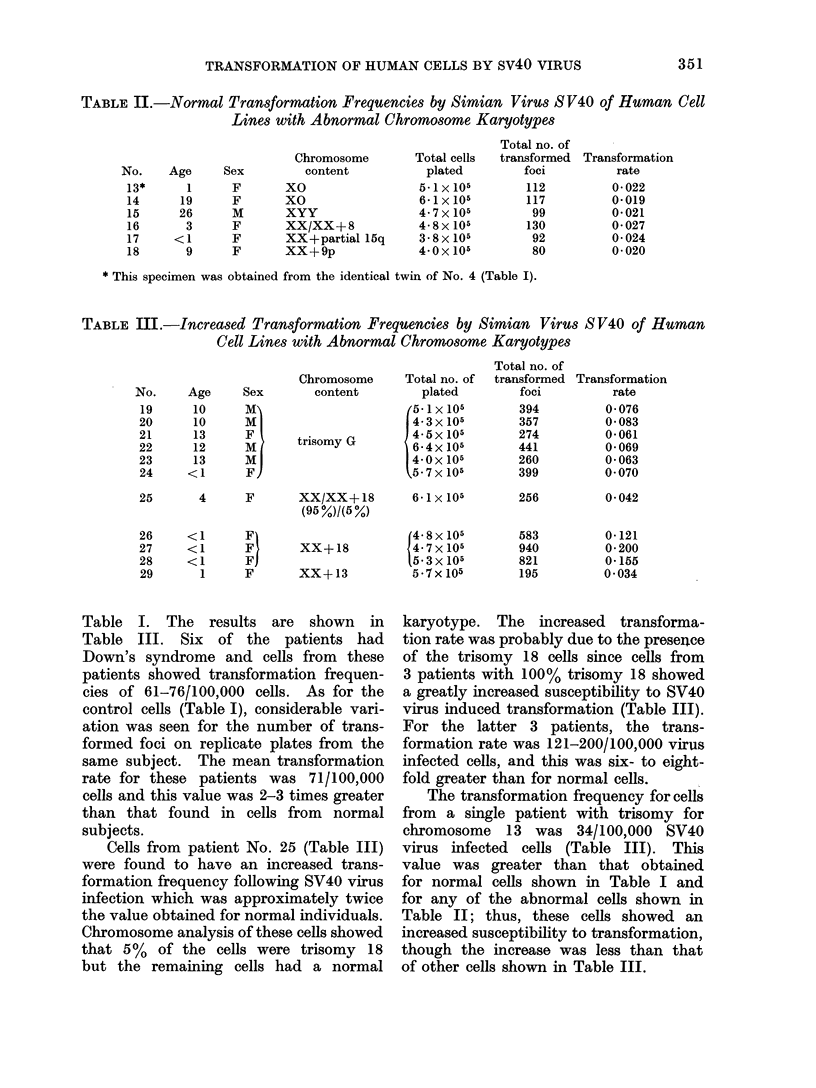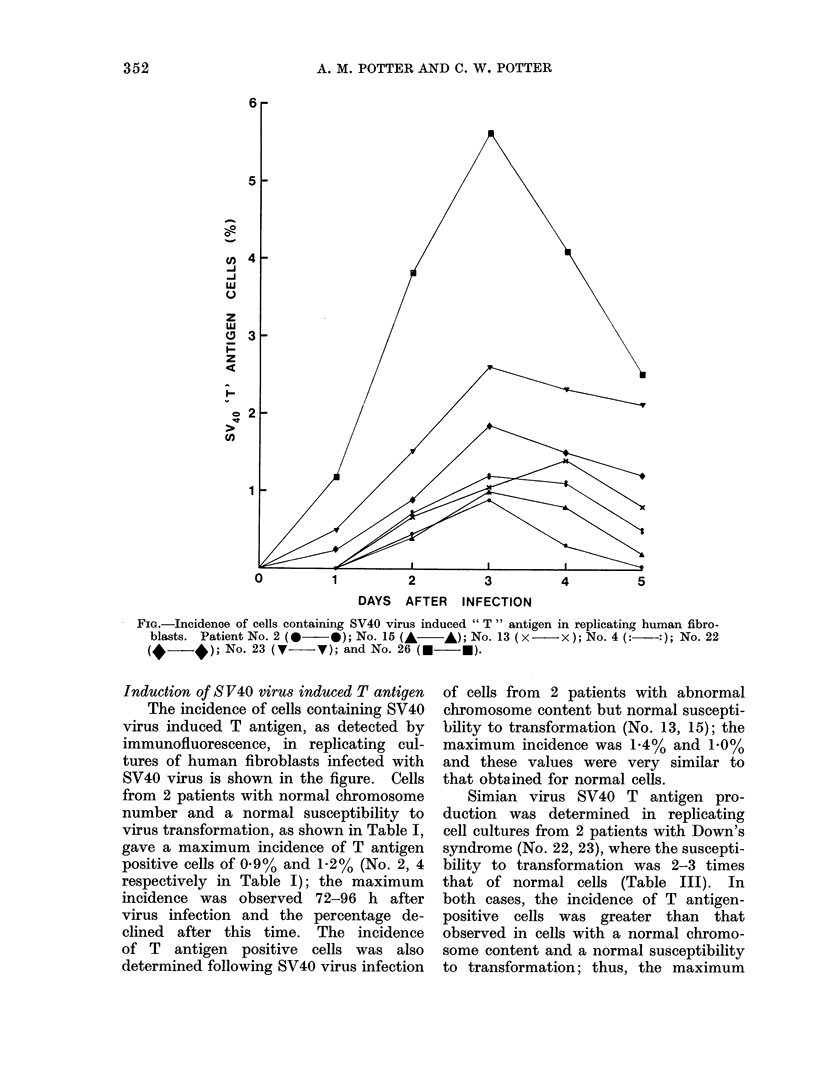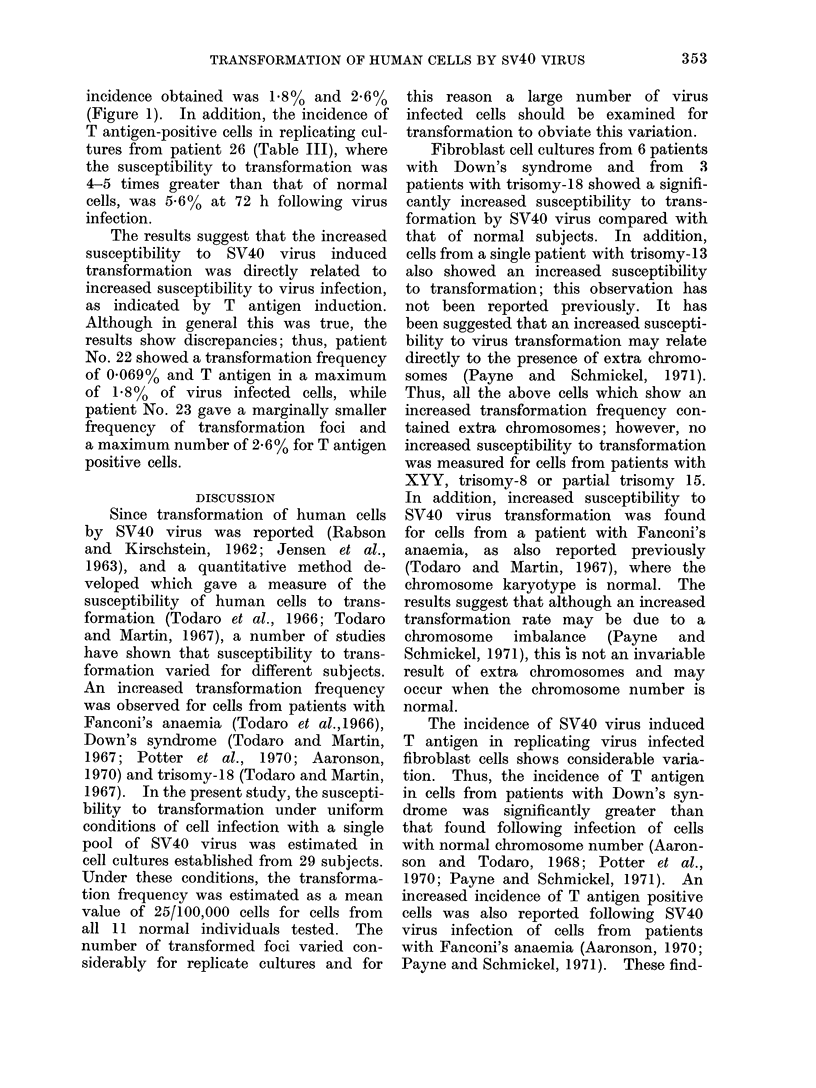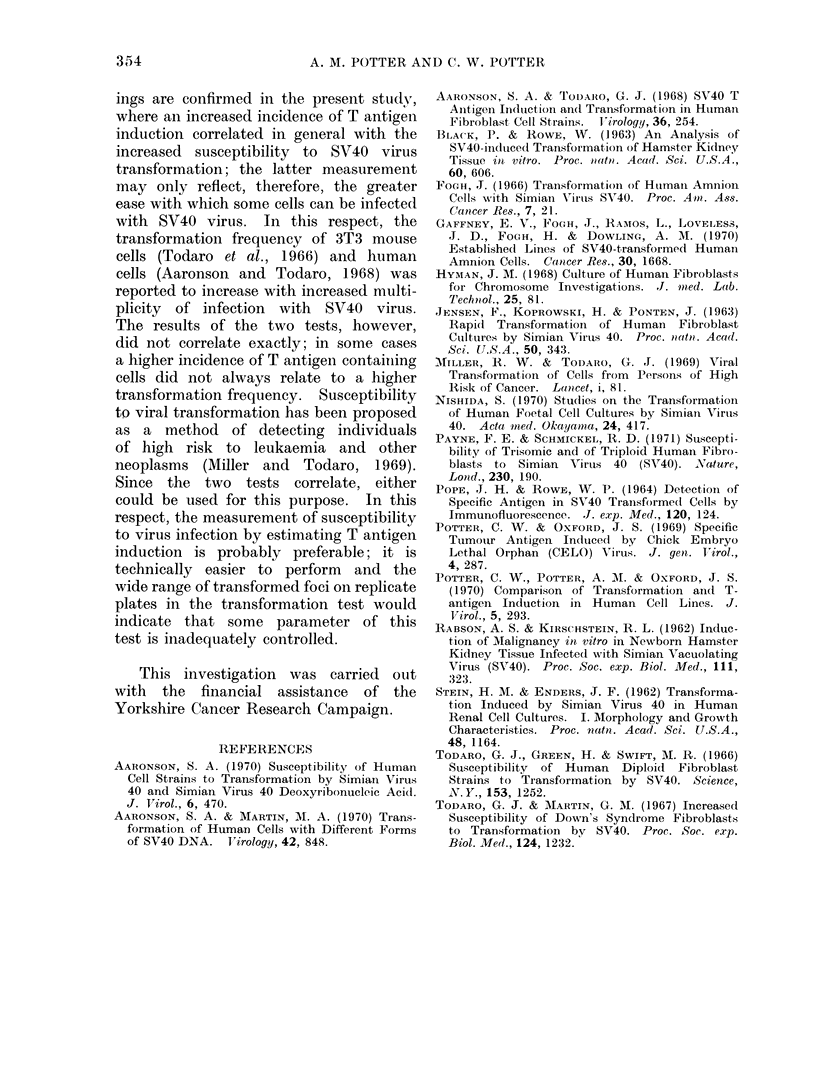# Transformation of human cells by SV40 virus.

**DOI:** 10.1038/bjc.1975.69

**Published:** 1975-03

**Authors:** A. M. Potter, C. W. Potter

## Abstract

Fibroblast cultures were prepared from skin biopsies from 29 patients and tested for their susceptibility to transformation by simian virus SV40. Cells with a normal chromosome complement showed a mean transformation frequency of 25/106 cells but for cells from a single patient with Fanconi's anaemia, the value was 152/106 cells. An increased susceptibility to transformation was observed for cells from 6 patients with Down's syndrome 3 patients with trisomy 18, a patient with trisomy 18 for 5% of cells and a patient with trisomy 13. No increased susceptibility to transformation was found for cells with a chromosome complement of XO, XXY, XX/XX + 8, XX + partial 15q or XX + 9p. The susceptiability to transformation was related to susceptibility to SV40 virus infection, as measured by the number of infected cells which contained SV40 virus induced T antigen. This latter test was technically easier to perform and could serve to detect persons of increased susceptiability to transformation, since this may indicate an increased risk of natural malignant disease.


*Br. J. Cancer* (1975) **31**, 348

### TRANSFORMATION OF HUMAN CELLS BY SV40 VIRUS

A. M. POTTER AND C. W. POTTER

*From the Centre for Human Genetics, Manchester Road, Sheffield, and the Academic Division of Pathology (Virology), University of Sheffield Medical School, Sheffield*

Received 19 November 1974. Accepted 5 December 1974

**Summary.—Fibroblast cultures were prepared from skin biopsies from 29 patients and tested for their susceptibility to transformation by simian virus SV40. Cells with a normal chromosome complement showed a mean transformation frequency of 25/10$^6$ cells but for cells from a single patient with Fanconi's anaemia, the value was 152/10$^6$ cells. An increased susceptibility to transformation was observed for cells from 6 patients with Down's syndrome, 3 patients with trisomy 18, a patient with trisomy 18 for 5% of cells and a patient with trisomy 13. No increased susceptibility to transformation was found for cells with a chromosome complement of XO, XXY, XX/XX + 8, XX + partial 15q or XX + 9p. The susceptibility to transformation was related to susceptibility to SV40 virus infection, as measured by the number of infected cells which contained SV40 virus induced T antigen. This latter test was technically easier to perform and could serve to detect persons of increased susceptibility to transformation, since this may indicate an increased risk of natural malignant disease.**

SIMIAN virus SV40 has been shown to transform cells from a variety of animals and from man (Rabson and Kirschstein, 1962; Stein and Enders, 1962; Jensen, Koprowski and Ponten, 1963; Black and Rowe, 1963; Gaffney *et al.*, 1970). The transformation of human cells has been reported for *in vitro* cultures established from a number of different tissues (Rabson and Kirshstein, 1962; Jensen *et al.*, 1963; Fogh, 1966; Nishida, 1970); in particular, cell cultures derived from small skin samples have been transformed by SV40 virus and this transformation can be accurately quantitated (Aaronson and Todaro, 1968; Aaronson and Martin, 1970; Potter, Potter and Oxford, 1970). In these studies, cultures of skin fibroblast cells from different subjects were found to vary significantly in susceptibility to transformation. Thus, cells from patients with Down's syndrome (Todaro, Green and Swift, 1966; Potter *et al.*, 1970) Fanconi's anaemia (Todaro *et al.*, 1966) were more susceptible to transformation by SV40 virus than cells from normal subjects; both groups of patients suffer a higher incidence of neoplastic disease than normal persons (Miller and Todaro, 1969).

In the present study, skin fibroblast cells from patients with a variety of chromosome aberrations were examined to determine their susceptibility to transformation by SV40 virus. The study was an extension of earlier observations which included the suggestion that cells containing extra chromosomes may be more susceptible to SV40 virus transformation than cells with a normal karyotype (Payne and Schmickel, 1971). In each case, a tissue culture was established from a skin biopsy and examined for chromosome content and susceptibility to transformation. In addition, the susceptibility of cells to virus infection was determined by measuring the presence of specific T antigen induced following infection with SV40 virus (Pope and Rowe, 1964).

### MATERIALS AND METHODS

*Cell cultures.*—Human fibroblast cultures were prepared from skin samples approximately 5 mm in diameter. The skin biopsy was cut into small pieces which were anchored in a glass vessel with human plasma clots (Hyman, 1968). The cultures were grown in Eagle's based medium (EBM) containing 10% foetal bovine serum (Flow Laboratories, Irvine, Scotland) and 10% tryptose phosphate broth and antibiotics (100 u/ml of penicillin and 100 μg/ml of streptomycin). The medium was changed twice weekly and confluent monolayers were obtained after 14–30 days incubation. Monolayer cultures were split at 7 day intervals and the chromosome content and susceptibility to SV40 virus transformation and T antigen induction was measured between the 4th to 10th *in vitro* passage.

African green monkey kidney cells (AGMK) were obtained as a cell suspension from Flow Laboratories Inc. and were cultured in medium 199 containing 5% inactivated calf sera, 0·44 μg/l sodium bicarbonate and antibiotics. These cells were maintained on the same medium adjusted to contain 2% calf serum.

*Chromosome analysis.*—Confluent monolayer cultures of skin fibroblasts were removed from culture vessels with 0·25% trypsin and inoculated into further vessels; the cells were suspended in medium 199 containing 20% foetal bovine serum and 20% tryptose phosphate broth and seeded at a concentration that gave 50% confluent growth after 36 h incubation at 37°C. After this time, Colcemid (GIBCO, Paisley, Scotland) was added to a final concentration of 0·02 μg/ml. After incubation at 37°C for 4–6 h, the cells were washed with phosphate buffered saline, detached from the culture vessels with 0·25% trypsin and swollen by the addition of an equal volume of distilled water. The cells were removed by centrifugation, fixed in acetic acid-ethyl alcohol (1 : 3), spread on grease-free, wet, ice cold slides and stained with 2% aceto-orcein. Chromosome counts were carried out on 30 well spread metaphase figures and a further 4 plates were selected for karyotype analysis.

*Viruses.*—Primary monolayers of AGMK cells in maintenance medium were infected with SV40 virus at a concentration of 0·1 $TCD_{50}$/cell. When cytopathic effect was complete, the cultures were frozen and thawed twice (−80°C/22°C) and centrifuged at 2000 *g* for 20 min to remove cell debris. The supernatant fluid containing $10^{8·3}$ $TCD_{50}$/ml of virus was stored at −80°C in 5–10 ml aliquots.

*Transformation studies.*—The transformation frequency of the human cell cultures was determined at the 4th–10th *in vitro* passage by the technique of Todaro *et al.* (1966). Ten to 20 cultures were used for each test and the results were expressed as the percentage of SV40 virus infected cells forming transformed foci; this assumed that each focus derived from a single transformed cell. Uninfected cultures were incubated in parallel to test for spontaneous transformation, but this was never observed.

*T antigen induction.*—Semi-confluent cultures of human fibroblast cells were infected with SV40 virus at a concentration of 1–2 $TCD_{50}$/cell and after 18 h incubation on growth medium the cells were removed with 0·25% trypsin, diluted 1 : 3 in growth medium containing 0·5% rabbit anti-SV40 virus serum (neutralizing titre 1 : 512), and seeded into petri dishes containing cover slips. The cells were incubated in growth medium in an atmosphere of 5% $CO_2$ in air. At intervals after seeding, coverslips were removed, washed in cold acetone (−20°C), air-dried and stored at −80°C.

The cover slip preparations were examined for SV40 virus induced T antigen by the indirect immunofluorescence technique (Pope and Rowe, 1964). The slips were thawed and stained with serum from hamsters bearing large, transplanted SV40 virus induced tumours; this serum had a complement fixing titre of 1 : 80 when titrated against tumour antigen prepared from homologous tumour tissue (Potter and Oxford, 1969). The slides were rinsed and stained with fluorescin labelled goat anti-hamster serum (Nordic Diagnostics, Tilburg, Netherlands) and viewed with a Gillett and Sibert conference microscope with an iodine-quartz light source. The percentage of cells with nuclei staining for T antigen was estimated from the observation of 2–4 × $10^3$ cells.

### RESULTS

### *Transformation of human fibroblasts with normal chromosome complement*

The incidence of transformed foci following infection with SV40 virus was

TABLE I.—*Transformation Frequencies by Simian Virus SV40 of Human Cell Lines from Subjects with Normal Chromosome Karyotypes*

| No. | Age (y) | Sex | Total infected cells plated | Total no. of transformed cells | Transformation rate |
|---|---|---|---|---|---|
| 1 | <1 | M | $5 \cdot 4 \times 10^5$ | 113 | 0·021 |
| 2 | <1 | M | $4 \cdot 4 \times 10^5$ | 106 | 0·024 |
| 3 | 26 | F | $7 \cdot 2 \times 10^5$ | 186 | 0·026 |
| 4 | 1 | F | $4 \cdot 8 \times 10^5$ | 101 | 0·021 |
| 5 | 27 | F | $5 \cdot 3 \times 10^5$ | 141 | 0·027 |
| 6 | 25 | M | $6 \cdot 1 \times 10^5$ | 183 | 0·030 |
| 7 | 4 | M | $6 \cdot 1 \times 10^5$ | 110 | 0·018 |
| 8 | 20 | M | $4 \cdot 1 \times 10^5$ | 97 | 0·024 |
| 9 | 35 | F | $1 \cdot 0 \times 10^6$ | 278 | 0·028 |
| 10 | 37 | M | $5 \cdot 1 \times 10^5$ | 134 | 0·026 |
| 11 | 2 | M | $6 \cdot 0 \times 10^5$ | 909 | 0·152 |
| 12 | 25 | F | $4 \cdot 7 \times 10^5$ | 130 | 0·028 |

determined for fibroblast cultures established from skin biopsies from 12 subjects whose cells had a normal chromosome content. The results are shown in Table I. For subjects 1–10 and 12 the transformation frequency was very similar; the rate was estimated to vary from 21–30/100,000 SV40 virus infected cells. No difference was observed in the transformation frequency of cells from male or female subjects. For the 4 subjects aged 4 years or less, the transformation rate was 18–25/100,000 cells (mean $210/10^6$ cells) and for the older subjects (mean age 28 years) the rate was 24–30 cells/100,000 (mean $264/10^6$ cells). This result suggested that cells from older subjects may be more susceptible to transformation by SV40 virus than cells from young children. However, although the above figure considers only the total number of transformed foci, there was considerable variation in the number of transformed foci seen in replicate cultures from the same subject and from different subjects. For this reason the statistical significance (correlation coefficient = 0·76 for q.d.f.; $P$ = 1·0–0·1%) for the difference in susceptibility to transformation for subjects of different age should not be considered reliable. The fibroblast cell culture No. 11 (Table I) was from a patient with Fanconi's anaemia; these cells showed a transformation frequency of $152/10^6$ cells, and this value was approximately six-fold greater than the mean value of $25/10^6$ cells obtained for the other 11 cases.

*Transformation of human fibroblasts with abnormal chromosome complement*

(a) *Normal transformation rate.*—Fibroblast cell cultures from 6 patients were found to have abnormal chromosome karyotypes but normal transformation frequencies were observed following infection with SV40 virus. The results are shown in Table II. Two of the patients had Turner's syndrome (XO) and one patient had an extra Y chromosome (XYY). Three further patients had extra chromosome material: one patient was partial trisomy for short arm 9 (XX9p+), one patient was trisomy for partial long arms 15 (XX15q+) and the remaining patient was a mosaic for trisomy 8 (XX/XX+8). The transformation frequency for these 6 patients varied from 19 to 27/100,000 SV40 virus infected cells, and the mean value was $225/10^6$ cells. These transformation frequencies were within the range of normal values determined in Table I.

(b) *Abnormal transformation rates.*—Fibroblast cells from 11 individuals contained an abnormal chromosome karyotype and in each case the transformation frequency following infection with SV40 virus was found to be significantly higher than that of cells from normal individuals, as established from subjects shown in

TABLE II.—*Normal Transformation Frequencies by Simian Virus SV40 of Human Cell Lines with Abnormal Chromosome Karyotypes*

| No. | Age | Sex | Chromosome content | Total cells plated | Total no. of transformed foci | Transformation rate |
|---|---|---|---|---|---|---|
| 13* | 1 | F | XO | $5 \cdot 1 \times 10^5$ | 112 | 0·022 |
| 14 | 19 | F | XO | $6 \cdot 1 \times 10^5$ | 117 | 0·019 |
| 15 | 26 | M | XYY | $4 \cdot 7 \times 10^5$ | 99 | 0·021 |
| 16 | 3 | F | XX/XX+8 | $4 \cdot 8 \times 10^5$ | 130 | 0·027 |
| 17 | <1 | F | XX+partial 15q | $3 \cdot 8 \times 10^5$ | 92 | 0·024 |
| 18 | 9 | F | XX+9p | $4 \cdot 0 \times 10^5$ | 80 | 0·020 |

* This specimen was obtained from the identical twin of No. 4 (Table I).

TABLE III.—*Increased Transformation Frequencies by Simian Virus SV40 of Human Cell Lines with Abnormal Chromosome Karyotypes*

| No. | Age | Sex | Chromosome content | Total no. of plated | Total no. of transformed foci | Transformation rate |
|---|---|---|---|---|---|---|
| 19 | 10 | M | trisomy G | $5 \cdot 1 \times 10^5$ | 394 | 0·076 |
| 20 | 10 | M | trisomy G | $4 \cdot 3 \times 10^5$ | 357 | 0·083 |
| 21 | 13 | F | trisomy G | $4 \cdot 5 \times 10^5$ | 274 | 0·061 |
| 22 | 12 | M | trisomy G | $6 \cdot 4 \times 10^5$ | 441 | 0·069 |
| 23 | 13 | M | trisomy G | $4 \cdot 0 \times 10^5$ | 260 | 0·063 |
| 24 | <1 | F | trisomy G | $5 \cdot 7 \times 10^5$ | 399 | 0·070 |
| 25 | 4 | F | XX/XX+18 (95%)/(5%) | $6 \cdot 1 \times 10^5$ | 256 | 0·042 |
| 26 | <1 | F | XX+18 | $4 \cdot 8 \times 10^5$ | 583 | 0·121 |
| 27 | <1 | F | XX+18 | $4 \cdot 7 \times 10^5$ | 940 | 0·200 |
| 28 | <1 | F | XX+18 | $5 \cdot 3 \times 10^5$ | 821 | 0·155 |
| 29 | 1 | F | XX+13 | $5 \cdot 7 \times 10^5$ | 195 | 0·034 |

Table I. The results are shown in Table III. Six of the patients had Down's syndrome and cells from these patients showed transformation frequencies of 61–76/100,000 cells. As for the control cells (Table I), considerable variation was seen for the number of transformed foci on replicate plates from the same subject. The mean transformation rate for these patients was 71/100,000 cells and this value was 2–3 times greater than that found in cells from normal subjects.

Cells from patient No. 25 (Table III) were found to have an increased transformation frequency following SV40 virus infection which was approximately twice the value obtained for normal individuals. Chromosome analysis of these cells showed that 5% of the cells were trisomy 18 but the remaining cells had a normal karyotype. The increased transformation rate was probably due to the presence of the trisomy 18 cells since cells from 3 patients with 100% trisomy 18 showed a greatly increased susceptibility to SV40 virus induced transformation (Table III). For the latter 3 patients, the transformation rate was 121–200/100,000 virus infected cells, and this was six- to eight-fold greater than for normal cells.

The transformation frequency for cells from a single patient with trisomy for chromosome 13 was 34/100,000 SV40 virus infected cells (Table III). This value was greater than that obtained for normal cells shown in Table I and for any of the abnormal cells shown in Table II; thus, these cells showed an increased susceptibility to transformation, though the increase was less than that of other cells shown in Table III.

FIG.—Incidence of cells containing SV40 virus induced " T " antigen in replicating human fibroblasts. Patient No. 2 (●——●); No. 15 (▲——▲); No. 13 (×——×); No. 4 (:——:); No. 22 (◆——◆); No. 23 (▼——▼); and No. 26 (■——■).

*Induction of SV40 virus induced T antigen*

The incidence of cells containing SV40 virus induced T antigen, as detected by immunofluorescence, in replicating cultures of human fibroblasts infected with SV40 virus is shown in the figure. Cells from 2 patients with normal chromosome number and a normal susceptibility to virus transformation, as shown in Table I, gave a maximum incidence of T antigen positive cells of 0·9% and 1·2% (No. 2, 4 respectively in Table I); the maximum incidence was observed 72–96 h after virus infection and the percentage declined after this time. The incidence of T antigen positive cells was also determined following SV40 virus infection of cells from 2 patients with abnormal chromosome content but normal susceptibility to transformation (No. 13, 15); the maximum incidence was 1·4% and 1·0% and these values were very similar to that obtained for normal cells.

Simian virus SV40 T antigen production was determined in replicating cell cultures from 2 patients with Down's syndrome (No. 22, 23), where the susceptibility to transformation was 2–3 times that of normal cells (Table III). In both cases, the incidence of T antigen-positive cells was greater than that observed in cells with a normal chromosome content and a normal susceptibility to transformation; thus, the maximum incidence obtained was 1·8% and 2·6% (Figure 1). In addition, the incidence of T antigen-positive cells in replicating cultures from patient 26 (Table III), where the susceptibility to transformation was 4–5 times greater than that of normal cells, was 5·6% at 72 h following virus infection.

The results suggest that the increased susceptibility to SV40 virus induced transformation was directly related to increased susceptibility to virus infection, as indicated by T antigen induction. Although in general this was true, the results show discrepancies; thus, patient No. 22 showed a transformation frequency of 0·069% and T antigen in a maximum of 1·8% of virus infected cells, while patient No. 23 gave a marginally smaller frequency of transformation foci and a maximum number of 2·6% for T antigen positive cells.

### DISCUSSION

Since transformation of human cells by SV40 virus was reported (Rabson and Kirschstein, 1962; Jensen *et al.*, 1963), and a quantitative method developed which gave a measure of the susceptibility of human cells to transformation (Todaro *et al.*, 1966; Todaro and Martin, 1967), a number of studies have shown that susceptibility to transformation varied for different subjects. An increased transformation frequency was observed for cells from patients with Fanconi's anaemia (Todaro *et al.*,1966), Down's syndrome (Todaro and Martin, 1967; Potter *et al.*, 1970; Aaronson, 1970) and trisomy-18 (Todaro and Martin, 1967). In the present study, the susceptibility to transformation under uniform conditions of cell infection with a single pool of SV40 virus was estimated in cell cultures established from 29 subjects. Under these conditions, the transformation frequency was estimated as a mean value of 25/100,000 cells for cells from all 11 normal individuals tested. The number of transformed foci varied considerably for replicate cultures and for this reason a large number of virus infected cells should be examined for transformation to obviate this variation.

Fibroblast cell cultures from 6 patients with Down's syndrome and from 3 patients with trisomy-18 showed a significantly increased susceptibility to transformation by SV40 virus compared with that of normal subjects. In addition, cells from a single patient with trisomy-13 also showed an increased susceptibility to transformation; this observation has not been reported previously. It has been suggested that an increased susceptibility to virus transformation may relate directly to the presence of extra chromosomes (Payne and Schmickel, 1971). Thus, all the above cells which show an increased transformation frequency contained extra chromosomes; however, no increased susceptibility to transformation was measured for cells from patients with XYY, trisomy-8 or partial trisomy 15. In addition, increased susceptibility to SV40 virus transformation was found for cells from a patient with Fanconi's anaemia, as also reported previously (Todaro and Martin, 1967), where the chromosome karyotype is normal. The results suggest that although an increased transformation rate may be due to a chromosome imbalance (Payne and Schmickel, 1971), this is not an invariable result of extra chromosomes and may occur when the chromosome number is normal.

The incidence of SV40 virus induced T antigen in replicating virus infected fibroblast cells shows considerable variation. Thus, the incidence of T antigen in cells from patients with Down's syndrome was significantly greater than that found following infection of cells with normal chromosome number (Aaronson and Todaro, 1968; Potter *et al.*, 1970; Payne and Schmickel, 1971). An increased incidence of T antigen positive cells was also reported following SV40 virus infection of cells from patients with Fanconi's anaemia (Aaronson, 1970; Payne and Schmickel, 1971). These findings are confirmed in the present study, where an increased incidence of T antigen induction correlated in general with the increased susceptibility to SV40 virus transformation; the latter measurement may only reflect, therefore, the greater ease with which some cells can be infected with SV40 virus. In this respect, the transformation frequency of 3T3 mouse cells (Todaro *et al.*, 1966) and human cells (Aaronson and Todaro, 1968) was reported to increase with increased multiplicity of infection with SV40 virus. The results of the two tests, however, did not correlate exactly; in some cases a higher incidence of T antigen containing cells did not always relate to a higher transformation frequency. Susceptibility to viral transformation has been proposed as a method of detecting individuals of high risk to leukaemia and other neoplasms (Miller and Todaro, 1969). Since the two tests correlate, either could be used for this purpose. In this respect, the measurement of susceptibility to virus infection by estimating T antigen induction is probably preferable; it is technically easier to perform and the wide range of transformed foci on replicate plates in the transformation test would indicate that some parameter of this test is inadequately controlled.

This investigation was carried out with the financial assistance of the Yorkshire Cancer Research Campaign.

### REFERENCES

AARONSON, S. A. (1970) Susceptibility of Human Cell Strains to Transformation by Simian Virus 40 and Simian Virus 40 Deoxyribonucleic Acid. *J. Virol.*, **6**, 470.

AARONSON, S. A. & MARTIN, M. A. (1970) Transformation of Human Cells with Different Forms of SV40 DNA. *Virology*, **42**, 848.

AARONSON, S. A. & TODARO, G. J. (1968) SV40 T Antigen Induction and Transformation in Human Fibroblast Cell Strains. *Virology*, **36**, 254.

BLACK, P. & ROWE, W. (1963) An Analysis of SV40-induced Transformation of Hamster Kidney Tissue *in vitro*. *Proc. natn. Acad. Sci. U.S.A.*, **60**, 606.

FOGH, J. (1966) Transformation of Human Amnion Cells with Simian Virus SV40. *Proc. Am. Ass. Cancer Res.*, **7**, 21.

GAFFNEY, E. V., FOGH, J., RAMOS, L., LOVELESS, J. D., FOGH, H. & DOWLING, A. M. (1970) Established Lines of SV40-transformed Human Amnion Cells. *Cancer Res.*, **30**, 1668.

HYMAN, J. M. (1968) Culture of Human Fibroblasts for Chromosome Investigations. *J. med. Lab. Technol.*, **25**, 81.

JENSEN, F., KOPROWSKI, H. & PONTEN, J. (1963) Rapid Transformation of Human Fibroblast Cultures by Simian Virus 40. *Proc. natn. Acad. Sci. U.S.A.*, **50**, 343.

MILLER, R. W. & TODARO, G. J. (1969) Viral Transformation of Cells from Persons of High Risk of Cancer. *Lancet*, i, 81.

NISHIDA, S. (1970) Studies on the Transformation of Human Foetal Cell Cultures by Simian Virus 40. *Acta med. Okayama*, **24**, 417.

PAYNE, F. E. & SCHMICKEL, R. D. (1971) Susceptibility of Trisomic and of Triploid Human Fibroblasts to Simian Virus 40 (SV40). *Nature, Lond.*, **230**, 190.

POPE, J. H. & ROWE, W. P. (1964) Detection of Specific Antigen in SV40 Transformed Cells by Immunofluorescence. *J. exp. Med.*, **120**, 124.

POTTER, C. W. & OXFORD, J. S. (1969) Specific Tumour Antigen Induced by Chick Embryo Lethal Orphan (CELO) Virus. *J. gen. Virol.*, **4**, 287.

POTTER, C. W., POTTER, A. M. & OXFORD, J. S. (1970) Comparison of Transformation and T-antigen Induction in Human Cell Lines. *J. Virol.*, **5**, 293.

RABSON, A. S. & KIRSCHSTEIN, R. L. (1962) Induction of Malignancy *in vitro* in Newborn Hamster Kidney Tissue Infected with Simian Vacuolating Virus (SV40). *Proc. Soc. exp. Biol. Med.*, **111**, 323.

STEIN, H. M. & ENDERS, J. F. (1962) Transformation Induced by Simian Virus 40 in Human Renal Cell Cultures. I. Morphology and Growth Characteristics. *Proc. natn. Acad. Sci. U.S.A.*, **48**, 1164.

TODARO, G. J., GREEN, H. & SWIFT, M. R. (1966) Susceptibility of Human Diploid Fibroblast Strains to Transformation by SV40. *Science, N.Y.*, **153**, 1252.

TODARO, G. J. & MARTIN, G. M. (1967) Increased Susceptibility of Down's Syndrome Fibroblasts to Transformation by SV40. *Proc. Soc. exp. Biol. Med.*, **124**, 1232.